# Brucellosis in Kazakhstan: Knowledge, Attitudes, and Practices Among Smallholder Farmers and Veterinary Specialists

**DOI:** 10.3390/vetsci13020191

**Published:** 2026-02-14

**Authors:** Spandiyar Tursunkulov, Faruza Zakirova, Zamzagul Moldakhmetova, Alexandra Tegza, Zaure Sayakova, Nurzhan Abekeshev, Alim Bizhanov, Assiya Mussayeva, Serik Kanatbayev, Gulnur Admanova, Nurkuisa Rametov, Temirlan Bakishev, Zhanar Bakisheva, Aigul Bulasheva, Akerke Temirova, Arman Issimov

**Affiliations:** 1 Institute of Animal Science and Veterinary Medicine, Saken Seifullin Kazakh Agrotechnical University, Astana 010000, Kazakhstan; 2 Department of Veterinary Medicine and Agrotechnology, Zhangir Khan West Kazakhstan Agrarian-Technical University, Oral 030000, Kazakhstan; 3 Department of Veterinary Sanitation, A. Baitursynov, Kostanay Regional University, Kostanay 110000, Kazakhstan; 4 Department of Bacteriology, Kazakh Research Veterinary Institute, Almaty 050016, Kazakhstan; 5 Kazakh Scientific Research Veterinary Institute, Branch “West Kazakhstan Scientific Research Veterinary Station”, Oral 030000, Kazakhstan; 6 Department of Biology, K. Zhubanov Aktobe Regional University, Aktobe 030000, Kazakhstan; 7 Department of Geospatial Engineering, Satpayev Kazakh National Research Technical University, Almaty 050016, Kazakhstan; 8 Department of Veterinary Science, Shakarim University, Semey 071412, Kazakhstan

**Keywords:** brucellosis, knowledge, attitudes, practices, Kazakhstan, cattle

## Abstract

Brucellosis continues to pose a substantial zoonotic risk in Kazakhstan; however, evidence describing the knowledge, attitudes, and practices (KAP) of cattle farmers and veterinary personnel remains limited. A cross-sectional study was undertaken between May and October 2024 across twelve administrative locations nationwide. Structured questionnaires were administered to 506 cattle farmers and 33 veterinary professionals, and the data were evaluated using descriptive analyses and univariable logistic regression. Awareness of brucellosis in cattle was relatively high among farmers, yet understanding of its implications for human health was markedly lower. In contrast, animal health workers demonstrated consistently higher levels of knowledge (OR: 12.6; 95% CI: 9.88–16.34; *p* < 0.001). Several practices associated with zoonotic transmission were commonly reported by farmers, including handling aborted materials without protective gloves, consumption of unpasteurised milk, and leaving reproductive tissues in grazing areas. Nevertheless, most farmers expressed readiness to adopt preventive measures, particularly cattle vaccination and the use of basic protective practices. These findings reveal important gaps between awareness and behavior that may contribute to continued transmission of brucellosis. Strengthening farmer education through locally tailored, One Health-based interventions offers a practical pathway to improving brucellosis control and enhancing cattle productivity in Kazakhstan.

## Simple Summary

Brucellosis is a disease that affects cattle and can also infect people, mainly through contact with animals or unsafe animal products. In Kazakhstan, brucellosis remains an important health and economic problem, but little is known about how well cattle farmers understand the disease or how their daily farming practices may influence its spread. This study examined farmers’ awareness of brucellosis, their attitudes toward prevention, and common cattle management practices, and compared these findings with the knowledge of veterinary professionals. Interviews showed that most farmers were aware of brucellosis in cattle, but many did not know that the disease can spread to humans. Several risky practices were common, such as handling birth materials without protection and leaving animal tissues in grazing areas. At the same time, most farmers expressed willingness to vaccinate cattle and use protective measures if these reduced disease risk. Improving farmer education, with support from veterinary professionals, may help reduce brucellosis transmission and improve both human health and livestock productivity in Kazakhstan.

## 1. Introduction

Brucellosis is a zoonotic infection affecting both animals and humans and represents a substantial public health and economic burden, particularly in developing countries [1]. In livestock populations, the disease is associated with impaired reproductive performance, increased rates of abortion, and reduced milk yield, leading to significant production losses [2]. Human infection most commonly results from the consumption of unpasteurised dairy products or direct exposure to contaminated animal tissues, including placental and aborted materials [3]. Individuals with occupational contact with livestock such as farmers, veterinarians, animal health technicians, and laboratory staff are at elevated risk of infection [4]. Clinically, human brucellosis is characterized by non-specific manifestations, including fever, malaise, myalgia, and arthralgia, which often complicate timely diagnosis. In advanced or untreated cases, serious complications such as infective endocarditis and osteomyelitis may develop [5].

Kazakhstan is the ninth largest country worldwide by land area and is characterized by substantial geographic heterogeneity, within which brucellosis cases have been reported among humans, livestock and wildlife populations [6,7]. Each year, over 1300 human brucellosis cases are officially reported, corresponding to an incidence rate of approximately 7.6 cases per 100,000 population. Serological surveillance indicates the presence of *Brucella*-specific antibodies in 0.6% of cattle and 0.4% of small ruminants [7,8]. Most farm animals are raised on privately operated smallholder farms managed by rural households. Livestock products obtained from these holdings are primarily intended for household consumption, although a proportion is distributed through informal marketing channels, including sales to intermediaries or direct exchange with neighbors, relatives, and local community members [9]. At the same time, the development of industrial-scale breeding programs continues to expand across the country [10,11]. Monitoring and control of livestock diseases are mandated responsibilities of state veterinary services. However, comprehensive national-level analyses remain limited, and available data are rarely examined in relation to geographic, social, or environmental determinants [12]. Compared with regions where zoonotic diseases are extensively documented, Kazakhstan remains underrepresented in the scientific literature, despite its location along major transcontinental trade routes connecting Europe and Asia [6]. Given the socio-cultural and economic significance of livestock, systematic investigation of brucellosis epidemiology in Kazakhstan is highly relevant.

Assessing knowledge, attitudes, and practices (KAPs) related to brucellosis is important for developing targeted strategies to prevent brucellosis outbreaks in livestock and to reduce the associated public health and economic impacts. Previous studies identified geographical factors and the molecular profile of *Brucella* strains circulating in Kazakhstan [9,13].

However, to our knowledge, no previous studies have systematically examined knowledge, attitudes, and practices related to brucellosis in Kazakhstan. Accordingly, the present study aims to assess KAPs concerning brucellosis among livestock farmers and veterinary professionals in selected regions of Kazakhstan.

## 2. Materials and Methods

### 2.1. Ethics

The protocol was approved by the Human Ethics Committee of the K. Zhubanov Aktobe Regional University (permit number: 09-08/09-2023). The study was conducted according to the principles of the Declaration of Helsinki (2013), and patient rights were observed. The purpose and methods of the current study were thoroughly explained to all participants, and informed oral consent was obtained and documented in the questionnaire.

### 2.2. Study Area and Data Collection

**Figure 1:**
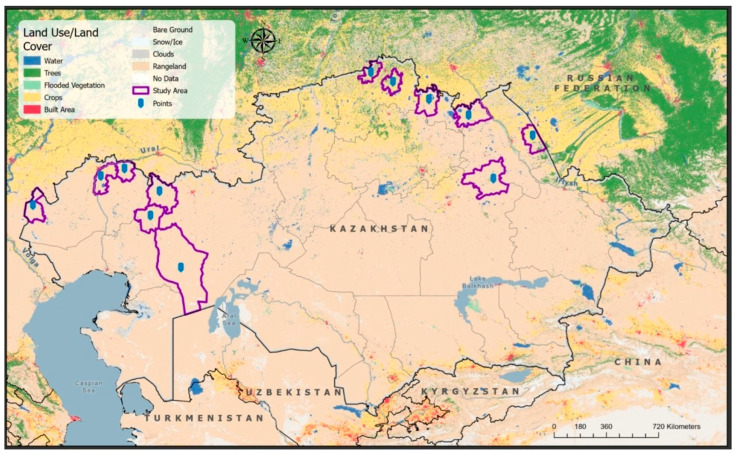
Selected districts of North Kazakhstan, Pavlodar, West Kazakhstan, and Aktobe regions for brucellosis KAP study. The purple-marked region highlights cattle distribution density. Cattle population data were obtained from the National Bureau of Statistics, Agency of the Republic of Kazakhstan for Strategic Planning and Reforms [14].

The study was carried out between May and October 2024 across twelve administrative locations within the North Kazakhstan, Pavlodar, West Kazakhstan, and Aktobe regions. Together, these regions encompass an area of approximately 779,000 km^2^ and are home to an estimated population of 3 million people and 30.2 million livestock [14]. The selected regions were chosen based on their high livestock density and the documented occurrence of brucellosis among agricultural animals, domestic species, and wildlife [9]. Spatial visualization and cartographic outputs were generated using ArcGIS Pro version 2.8 (ESRI, Redlands, CA, USA), a geographic information system (GIS) platform for spatial data analysis and mapping (Figure 1). All geographic datasets were projected using the World Geodetic System 1984 (WGS 84, Springfield, VA, USA), the standard global reference system for latitude and longitude coordinates [15]. Field-collected data were subsequently integrated with supplementary spatial layers, including Sentinel-2 satellite imagery (10 m resolution; Land Use/Land Cover), topographic features, and administrative boundaries [16].

Sample size estimation was performed following the approach outlined by Thrusfield [17]. An expected prevalence of 50%, a confidence level of 95%, and a precision of 5% were applied to ensure sufficient data collection on knowledge, attitudes, and practices related to brucellosis among farmers in the selected districts. Based on these parameters, the required sample size was calculated to be 506 respondents. Eligibility for participation required that respondents belong to households owning at least one bovine animal and be 20 years of age or older. All enrolled farmers completed interviews assessing their knowledge, attitudes, and practices related to brucellosis as a zoonotic infection. Participation was voluntary, and farmers were recruited based on their willingness to take part in the study. In instances where a selected farm owner declined participation, an alternative eligible farmer was recruited as a replacement. The overall participation rate reached 90%. Interviews were conducted on-site at the farms, and all interviewers received training in the administration of the survey instrument.

### 2.3. Questionnaire

The survey instrument for cattle farmers was initially designed in English and subsequently translated into local Kazakh and Russian languages. Prior to full deployment, the questionnaire was pilot-tested with 15 cattle farmers residing near the study locations, and minor revisions were made to enhance clarity of the questions. The finalized questionnaire consisted of 32 questions organized into four thematic sections: (i) participant sociodemographic characteristics, (ii) knowledge of brucellosis as a zoonotic disease, (iii) attitudes toward brucellosis, and (iv) self-reported practices associated with potential zoonotic transmission. In parallel with the farmer-based cross-sectional survey, an independent cross-sectional assessment was conducted among animal health personnel to evaluate their knowledge and professional practices related to brucellosis. Eligible participants included government-employed veterinarians and livestock technicians actively involved in providing animal health services to livestock. In accordance with District Veterinary Office (DVO) staffing regulations, each district is served by one veterinarian and at least two veterinary technicians. Overall, the survey included 12 veterinarians and 21 veterinary technicians.

### 2.4. Data Analysis

Collected data were entered into Microsoft Excel, cleaned, and analysed using the Python programming environment (Jupyter Notebook, version 3.9.7, San Francisco, CA, USA). Several demographic variables were recoded into categorical formats for analytical purposes, including age, educational attainment, and herd size. Categorical data were summarised using absolute and relative frequencies, while continuous variables, which showed non-normal distributions, were described using medians, interquartile ranges, and ranges. Associations between gender and engagement in cattle-related activities, as well as between herd size and management practices, were examined using univariable logistic regression models with adjustment for clustering. Associations between occupation (farmer vs. animal health worker) and knowledge of brucellosis in humans and cattle were assessed using similar univariable models. Effect estimates are presented as odds ratios with 95% confidence intervals, and a value of *p* ≤ 0.05 was designated statistically significant.

## 3. Results

### 3.1. Socio-Demographic Characteristics of Cattle Farmers

The demographic characteristics of farmers are summarized in Table 1. The median age of respondents was 52 years, while the median duration of involvement in cattle farming was 14 years. The majority of participants were male (72%; 364/506), and men were most frequently identified as the primary individuals responsible for cattle management within households (75%; 379/506). In addition to cattle, a substantial proportion of farmers reported involvement in the care of other animal species susceptible to brucellosis, including sheep (80.2%; 406/506) and goats (37.3%; 189/506). Participation in these activities was comparable between male and female farmers.

Cattle slaughter was reported primarily in connection with sociocultural practices, including funeral rites (45%; 228/506), ceremonial house rituals (61%; 309/506), and wedding celebrations (75%; 380/506). Engagement in cattle milking was rare, with only two respondents indicating prior involvement in this activity.

Several cattle-related activities demonstrated a strong association with male gender, particularly Cattle slaughter or carcass processing (OR: 13.4; 95% CI: 5.7–29.4; *p* = 0.002), cattle trading (OR: 3.2; 95% CI: 1.9–4.8; *p* = 0.003) and cleaning cattle manure (OR: 4.8; 95% CI: 2.6–6.4; *p* = 0.002). Male participants were also more frequently involved in assisting with calving or abortion events (OR: 3.6; 95% CI: 1.5–4.4; *p* = 0.214); however, the wide confidence interval indicates limited precision, precluding firm conclusions. Detailed results of the regression analyses are presented in Supplementary Table S1.

### 3.2. Cattle Herd Characteristics

With the exception of a single farm that maintained both Kazakh white-headed and crossbreed cattle, nearly all surveyed farms exclusively kept crossbreed cattle (97%; 491/506). The median herd size was seven animals per household. Fewer than half of respondents (43.4%; 220/506) reported keeping male cattle older than two years, whereas female cattle in the same age category were present in almost all households (96.8%; 490/506). During the preceding 12 months, farmers reported a median of two calving events. Approximately one-third of farmers (34.6%; 175/506) indicated a history of cattle abortions, with more than half of these farms reporting a higher frequency during the winter season (52.2%; 264/506). Among affected farms, the median number of abortions within the past year was one per farm. Only a limited proportion of farms (11.3%; 57/506) had received support from government veterinary or livestock technicians in the previous year.

Cattle management practices varied, with tethering being the most common approach (84.7%; 428/506), followed by free-roaming (64.4%; 326/506) and yarding or penning (30.6%; 155/506). Free-roaming was never practiced in isolation but was combined with other methods. Concerns regarding crop damage during the wet season and prevention of cattle theft at night were frequently cited as reasons for limiting exclusive free-roaming.

### 3.3. Knowledge and Attitudes of Farmers Regarding Brucellosis

**Figure 2:**
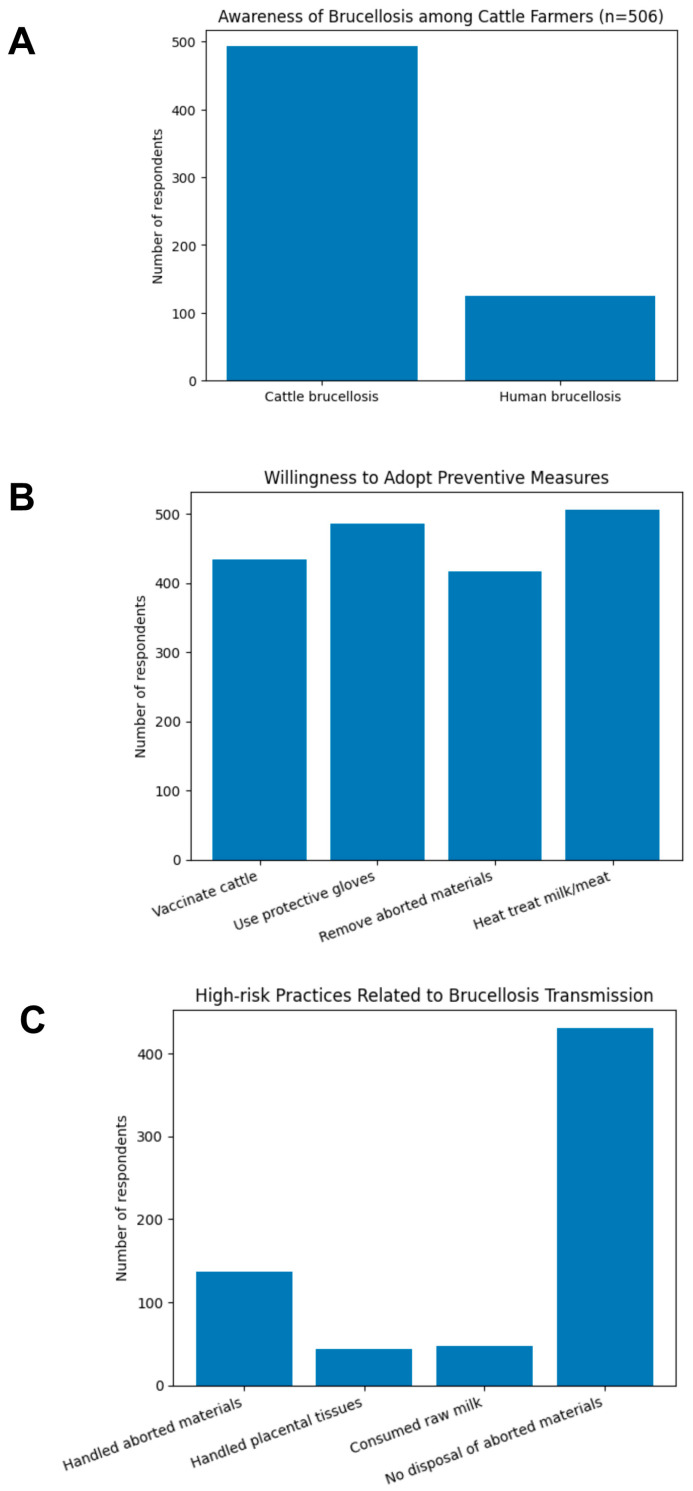
Awareness of brucellosis, risk-related behaviors, and prevention attitudes among cattle farmers. (**A**) Awareness of brucellosis in cattle and humans. (**B**) Selected practices associated with potential zoonotic transmission. (**C**) Attitudes toward key preventive measures.

The KAP of 506 cattle farmers in relation to brucellosis as a zoonotic and/or cattle disease are summarized in Table 2. Awareness of brucellosis in cattle was high among respondents, whereas awareness of human brucellosis was substantially lower (Figure 2A). Among those who acknowledged familiarity with brucellosis in humans, only two participants were able to correctly identify potential transmission pathways from cattle to humans. Village animal health practitioners were identified as the primary source of information on brucellosis for most farmers, while informal sources such as neighbours and friends were less frequently cited (Table 2). The vast majority of respondents indicated a willingness to use protective gloves when handling aborted materials or placental tissues if this measure were shown to reduce their risk of brucellosis (Figure 2B). Participants who expressed uncertainty or reluctance toward glove use cited limited access to protective equipment or the absence of prior illness despite not using gloves. Additionally, all respondents reported that they would ensure thorough cooking of cattle-derived products if this lowered the risk of zoonotic transmission of brucellosis (Figure 2B).

Among farmers interviewed, only six respondents attributed cattle abortions to infectious causes, while the majority (62%; 314/506) were unable to identify any specific cause. Some respondents attributed cattle abortion to factors such as injury or physical stress (n = 71), inadequate nutrition (n = 67), and poor care (n = 43). A high proportion of households expressed willingness to vaccinate their cattle (85.7%; 434/506) and to remove aborted materials from grazing areas (82.4%; 417/506) if these measures were shown to reduce brucellosis transmission. Households expressing uncertainty or reluctance toward vaccination or removal of aborted materials commonly cited the challenges associated with free-roaming cattle, including difficulties in animal restraint and detection of abortion events. Similarly, households unwilling to separate cattle reported insufficient feed resources and limited space as key constraints. Among the 104 households that explicitly opposed to get rid of (slaughter) infected cattle, the most frequently reported reasons included concerns about financial loss (n = 69).

### 3.4. Practices of Farmers Regarding Brucellosis

A small proportion of the respondents (9.3%; 47/506) reported consuming raw cow milk. Approximately 27% (137/506) of participants reported prior involvement in handling aborted fetuses or stillborn calves, with glove use reported by only one individual (Figure 2C). Handling of bovine placental membranes was uncommon, reported by 8.6% (43/506) of respondents, and protective glove use was again reported by only a single participant. Male participants demonstrated a higher likelihood than females of covering open cuts (OR: 4.3; 95% CI: 2.2–7.4; *p* = 0.022) and of handling aborted materials (OR: 2.4; 95% CI: 1.2–5.1; *p* = 0.013).

Most households (90.6%; 458/506) grazed their cattle on communal pasturelands rather than on privately owned land. Herd replacement was primarily achieved through on-farm rearing (92.0%; 466/506). Among households that introduced cattle from external sources (8.0%; 40/506), only one reported occasionally consulting government veterinary technicians to evaluate animal health prior introducing into the existing herd. During at least one calving event, the majority of households (77.0%; 390/506) reported taking no specific action to manage placental membranes. Of these, 390 respondents reported that placental tissues were consumed by cattle (47.3%; 184/390) or by dogs (23.6%; 92/390) in the field. Similarly, 85.1% (431/506) of households indicated that aborted materials were not disposed of on at least one occasion, with several reporting that such materials were consumed by cattle (19%; 82/431) or dogs (24.8%; 107/431).

### 3.5. Animal Health Practitioners

In total, 33 animal health practitioners were enrolled in the study, with their knowledge and practices summarized in Table 3. Relative to cattle farmers, animal health practitioners demonstrated higher awareness of brucellosis in humans (OR: 12.6; 95% CI: 9.88–16.34; *p* < 0.001). Detailed results of the regression analyses are presented in Supplementary Table S2.

## 4. Discussion

### 4.1. Knowledge of Brucellosis and Information Pathways Among Farmers

Understanding farmers’ knowledge of brucellosis and their primary sources of information is essential for designing effective disease control strategies. In the present study, the survey provided the first systematic insight into brucellosis-related awareness among cattle farmers and veterinary service providers in Kazakhstan. [18]. In the study area, the administered survey provided insights into the level of awareness and prevailing approaches of brucellosis among a selected group of farmers. Notably, this study offers the first practical assessment of KAPs related to brucellosis among livestock farmers and veterinary service providers in Kazakhstan. The findings indicated that nearly all interviewed respondents reported prior awareness of brucellosis. The findings of this study demonstrated that cattle farmers had limited knowledge of brucellosis in cattle, including awareness of clinical manifestations and transmission pathways. This observation aligns with reports from other Asian countries that have documented similarly low levels of brucellosis-related knowledge among farmers [19,20,21]. Insufficient understanding of brucellosis and other infectious diseases was further reflected in the absence of basic biosecurity measures, such as health screening and temporary separation of newly introduced cattle to reduce the risk of disease introduction, as observed in the present study. In contrast, animal health practitioners demonstrated substantially higher levels of brucellosis-related knowledge, with nearly all respondents accurately identifying transmission routes and control strategies. This difference likely reflects their formal professional training and participation in a government-led brucellosis training workshop conducted shortly before the survey. Similar patterns have been reported elsewhere, where animal health professionals exhibited greater awareness of brucellosis than livestock farmers [22]. Additionally, the present study demonstrates that local animal health professionals serve as the primary sources of information for farmers. These results further reinforce the view that effective farm biosecurity can be promoted through the enhancement of producers’ knowledge [23].

### 4.2. Attitudes Toward Brucellosis Prevention and the Knowledge–Practice Gap

Beyond awareness, farmers’ attitudes toward preventive measures play a critical role in determining the effectiveness of brucellosis control programmes. The present findings reveal a notable gap between farmers’ stated willingness to adopt preventive measures and their reported practices in daily cattle management. With respect to attitudes, most farmers indicated a willingness to use protective gloves to reduce the risk of transmission; however, actual use during the handling of aborted materials was uncommon. This discrepancy is likely attributable to the cost and limited availability of protective equipment in the study area. The high proportion of farmers expressing willingness to vaccinate their cattle (85.7%) suggests that vaccination campaigns may be effective, particularly if implemented during periods when cattle movement and free-ranging are limited. In contrast, willingness to slaughter infected animals as a disease control measure was markedly lower than for other interventions. This reluctance likely reflects the role of cattle as both an economic asset and a marker of social status within the community [24]. These findings indicate that test-and-slaughter strategies for brucellosis control are unlikely to achieve broad acceptance unless supported by adequate financial compensation, although the provision of such incentives often remains challenging in resource-limited countries [25,26].

### 4.3. High-Risk Farming Practices and Implications for Zoonotic Transmission

In addition to attitudes, several routine cattle management practices identified in this study may contribute to sustained transmission of brucellosis within cattle populations and increased zoonotic risk for humans. These include communal grazing systems [4], the co-rearing of multiple animal species within the same household [27], and the introduction of replacement cattle sourced from external farms [28]. Additionally, the feeding or exposure of dogs to birth or aborted materials represents a further risk, as dogs may function as mechanical vectors or reservoir hosts for *Brucella* spp. [29,30]. Furthermore, the widespread practice of leaving placental tissues or aborted materials unattended in grazing areas may exacerbate disease spread, given that reproductive tissues from infected cattle constitute a major source of environmental contamination and transmission [31]. With regard to practices associated with zoonotic transmission, the present study highlights multiple scenarios in which both cattle farmers and animal health workers in Kazakhstan may be exposed to brucellosis originating from cattle. Several well-established risk factors for human infection, including cattle slaughter [32], handling aborted materials or placental tissues without protective gloves [33], and the consumption of unpasteurised milk [34] were reported among study participants. Regarding herd demographics, cattle populations were predominantly composed of female animals, while cattle management activities were largely undertaken by male farmers, a pattern consistent with previous findings from Kazakhstan [12,35]. This study delineates specific cattle-related tasks that were primarily performed by men, including slaughtering, cattle trading, and the handling of aborted materials. Engagement in these activities may place male farmers at an elevated risk of exposure to brucellosis compared with their female counterparts.

There are several limitations to this study that should be acknowledged. The primary limitation of this study relates to the reliability of household-reported data. Participants may have introduced reporting bias through inaccurate or selective disclosure of information, including the potential overstatement of losses. Additionally, the phrasing of some questions in the attitude section of the surveys (e.g., Would you be willing to use protective gloves if doing so reduced your risk of acquiring brucellosis from cattle?) could have made it hard for the participants to answer negatively, potentially leading to a biased response. Future surveys could consider rephrasing such questions using more neutral language.

## 5. Conclusions

This study provides the first comprehensive assessment of knowledge, attitudes, and practices related to brucellosis in both cattle and humans in Kazakhstan. Awareness of brucellosis was generally low among livestock farmers but substantially higher among animal health professionals. Although knowledge gaps were evident, most farmers expressed a willingness to vaccinate their cattle, suggesting that vaccination programmes may be effective, particularly if implemented during periods when cattle movement is limited. Several practices associated with zoonotic transmission were identified, including cattle slaughter, handling of aborted or birthing materials with minimal protective measures, and consumption of unpasteurised milk. Addressing identified high-risk practices through targeted education and locally adapted, One Health-oriented control strategies offers a clear opportunity to reduce brucellosis transmission and improve livestock productivity in Kazakhstan.

## Figures and Tables

**Table 1:** Socio-demographic characteristics of participants (*n* = 506).

Variable/Category	Male (%) (n—409)	Female (%) (n—97)	Total (%) (n—506)
Education			
Primary	164 (40.1)	33 (34.0)	197 (39.0)
Secondary	143 (35)	42 (43.3)	185 (36.6)
Tertiary	102 (25)	22 (22.7)	124 (24.5)
Cattle management activities			
Feeding cattle	371 (93.2)	82 (85.4)	453 (89.5)
Cattle trading	297 (72.6)	57 (58.5)	354 (70.0)
Involvement in calving or abortion in cattle	63 (15.5)	9 (9.3)	72 (14.2)
Cattle slaughter or carcass processing	117 (28.5)	4 (4.2)	121 (24.0)
Milking cattle	0 (0.0)	11 (11.5)	11 (2.2)
Cleaning cattle manure	273 (66.7)	3 (2.7)	276 (54.5)
Management of other animals susceptible to brucellosis			
Sheep	334 (81.6)	72 (74.0)	406 (80.2)
Goat	160 (39.2)	29 (30.1)	189 (37.3)
Pig	11 (2.7)	0 (0.0)	11 (2.2)
None of the above	97 (23.6)	20 (21.1)	117 (23.1)

**Table 2:** Knowledge, attitude and practices of participants (*n* = 506) towards brucellosis.

Variables	Frequency (%)
Do you believe that diseases can be transmitted from cattle to humans?	
Yes	264 (52.2)
No	43 (8.5)
Not sure	199 (39.3)
Prior to this survey, were you aware of a disease known as brucellosis in cattle?	
Yes	493(97.5)
No	3 (0.6)
Not sure	10 (2.0)
Prior to this survey, were you aware of a disease known as brucellosis in humans?	
Yes	125(24.7)
No	35 (7.0)
Not sure	346 (68.4)
Source of information about brucellosis?	
Friends	49 (9.7)
Neighbours	101 (20.0)
Animal health practitioners	295 (58.3)
Media	61 (12.0)
In your opinion, what are the main causes of abortion in cattle?	
I don’t know	314 (62.0)
Injury or physical stress	76 (15.0)
Poor feeding	67 (13.3)
Poor care	43 (8.5)
Infectious diseases	6 (1.2)
Do you believe that diseases transmitted from cattle, such as brucellosis, can cause serious illness in you and your family?	
Yes	158 (31.2)
No	143 (28.3)
Not sure	205 (40.5)
Would you be willing to use protective gloves if doing so reduced your risk of acquiring brucellosis from cattle?	
Yes	485 (95.8)
No	8 (1.6)
Not sure	13 (2.6)
Would you be willing to ensure thorough cooking or appropriate heat treatment of cattle-derived products (milk, offal, and meat) if this reduced your risk of brucellosis transmission from cattle?	
Yes	506 (100.0)
No	0 (0.0)
Not sure	0 (0.0)
Would you be willing to vaccinate your cattle against brucellosis?	
Yes	434 (85.7)
No	18 (3.6)
Not sure	54 (10.6)
Do you remove aborted materials from grazing areas or yards/premises?	
Yes	417 (82.4)
No	89 (17.6)
Would you be willing to get rid of the cattle infected with brucellosis if this measure reduced further transmission of the disease?	
Yes	390 (77.1)
No	104 (20.5)
Not sure	12 (2.4)
What grazing areas are used for your cattle?	
Communal grazing	458 (90.6)
Own land	48 (9.4)
How do you replace your cattle herd?	
On-farm rearing	466 (92.0)
Purchasing or exchanging from other farms	40 (8.0)
Other	0 (0.0)
When in contact with cattle, do you take measures to protect hand wounds, such as covering cuts with a bandage or cloth?	
Yes	213 (42.1)
No	266 (52.6)
Not sure	27 (5.3)
Have you previously consumed raw or unprocessed milk or milk products from cattle?	
Yes	47 (9.3)
No	453 (89.5)
Not sure	6 (1.2)
Have you ever consumed raw or inadequately cooked meat from cattle?	
Yes	0 (0.0)
No	493 (97.4)
Not sure	13 (2.6)
Have you previously handled aborted fetal material or stillborn calves?	
Yes	137 (27.0)
No	369 (73.0)
Not sure	0 (0.0)
Have you ever handled placental tissues from cows during or after calving?	
Yes	43 (8.6)
No	463 (91.4)
Not sure	0 (0.0)
What do you do with placenta following calving?	
Nothing	390 (77.0)
Bury	38 (7.5)
Burn	17 (3.4)
Throw away	61 (12.1)
What do you do with aborted materials?	
Nothing	431 (85.1)
Bury	29 (5.8)
Burn	10 (2.0)
Throw away	36 (7.1)

**Table 3:** Knowledge and practices of animal health practitioners (n = 33) about brucellosis in the area studied.

Variables	Frequency (%)
Prior to this survey, were you aware of a disease known as brucellosis affecting cattle?	
Yes	33 (100)
No	0 (0.0)
Not sure	0 (0.0)
Which pathogen is responsible for causing brucellosis?	
Parasite	4 (71.5)
Bacteria	7 (19.0)
Virus	12 (9.5)
Do you know that cattle may acquire brucellosis through direct contact with infected animals?	
Yes	31 (94.0)
No	0 (0.0)
Not sure	2 (6.0)
Do you know that cattle may become infected with brucellosis through contact with, or ingestion of, aborted materials from infected animals?	
Yes	33 (100)
No	0 (0.0)
Not sure	0 (0.0)
Do you believe that grazing on contaminated pasture can lead to brucellosis infection in cattle?	
Yes	33 (100)
No	0 (0.0)
Not sure	0 (0.0)
Prior to this survey, were you aware of a disease known as brucellosis affecting humans?	
Yes	27 (81.8)
No	4 (12.1)
Not sure	2 (6.1)
Do you know the routes through which humans may acquire brucellosis from cattle?	
Yes	29 (87.9)
No	0 (0.0)
Not sure	4 (12.1)
Have you ever been involved in handling aborted fetuses or stillborn calves?	
Yes	28 (84.8)
No	5 (31.8)
Not sure	0 (15.2)
Have you ever handled placental tissues during or following calving?	
Yes	30(91.0)
No	3 (9.0)
Not sure	0 (0.0)

## Data Availability

The original contributions presented in this study are included in the article/Supplementary Material. Further inquiries can be directed to the corresponding authors.

## Supplementary Materials

The following supporting information can be downloaded at: https://www.mdpi.com/article/10.3390/vetsci13020191/s1, Table S1: Results from random effects regression analyses assessing associations between gender and cattle farming activities; Table S2: Results from regression analyses assessing associations between knowledge variables.
