# Supplementary material for: Brucellosis in Kazakhstan: Knowledge, Attitudes, and Practices Among Smallholder Farmers and Veterinary Specialists
Source: Vet Sci. 2026 Feb 14;13(2):191. doi: 10.3390/vetsci13020191 (PMC12944875; doi:10.3390/vetsci13020191)
Supplement: Supplementary file 1 [file vetsci-13-00191-s001.zip › vetsci-4122977-SM.pdf]

Supplementary Table S1. Results from random effects regression analyses assessing associations between gender and cattle farming activities reported by 506 cattle.

**Variables**	**Odd ratio (95% CI)**	**P- value**
Trading cattle		
Male	3.2 (1.9 - 4.8)	**0.003**
Female	Ref	
Cleaning cattle manure from pens/yards		
Male	4.8 (2.6 - 6.4)	**0.002**
Female	Ref	
Giving feed/water to cattle		
Male	1.2 (0.6 – 2.1)	0.133
Female	Ref	
Slaughtering/butchering cattle		
Male	13.4 (5.7 - 29.4)	**0.002**
Female	Ref	
Helping with birth and/or abortion in cattle		
Male	3.6 (1.5 - 4.4)	0.214
Female	Ref	

Supplementary Table S2. Results from regression analyses assessing associations between position and knowledge variables reported by 506 cattle farmers and 33 animal health practitioners.

**Question**	**Yes, %**	**Odd ratio (95% CI)**	**P- value**
Heard of brucellosis in humans before survey			
Farmer	24.7	Ref	<0.001
Technician	81.8	12.6 (9.88–16.34)	
